# Evidence for the embodiment of space perception: concurrent hand but not arm action moderates reachability and egocentric distance perception

**DOI:** 10.3389/fpsyg.2015.00862

**Published:** 2015-06-26

**Authors:** Stéphane Grade, Mauro Pesenti, Martin G. Edwards

**Affiliations:** ^1^Institut de Recherche en Sciences Psychologiques, Université Catholique de Louvain, Louvain-la-Neuve, Belgium; ^2^Institute of Neuroscience, Université Catholique de Louvain, Louvain-la-Neuve, Belgium

**Keywords:** reachability judgment, distance estimation, action simulation, dual-task, space perception

## Abstract

The perception of reachability (i.e., whether an object is within reach) relies on body representations and action simulation. Similarly, egocentric distance estimation (i.e., the perception of the distance an object is from the self) is thought to be partly derived from embodied action simulation. Although motor simulation is important for both, it is unclear whether the cognitive processes underlying these behaviors rely on the same motor processes. To investigate this, we measured the impact of a motor interference dual-task paradigm on reachability judgment and egocentric distance estimation, while allocentric length estimation (i.e., how distant two stimuli are from each other independent from the self) was used as a control task. Participants were required to make concurrent actions with either hand actions of foam ball grip squeezing or arm actions of weight lifting, or no concurrent actions. Results showed that concurrent squeeze actions significantly slowed response speed in the reachability judgment and egocentric distance estimation tasks, but that there was no impact of the concurrent actions on allocentric length estimation. Together, these results suggest that reachability and distance perception, both egocentric perspective tasks, and in contrast to the allocentric perspective task, involve action simulation cognitive processes. The results are discussed in terms of the implication of action simulation when evaluating the position of a target relative to the observer’s body, supporting an embodied view of spatial cognition.

## Introduction

Space perception arises from multimodal integration (Andersen et al., 1997). Some studies show that neurons are active for both tactile and visual stimulation within a delimited space surrounding and anchored to specific body parts (see Graziano et al., 1994; Gross and Graziano, 1995; Fadiga et al., 2000), while other studies indicate that space perception can be derived from sensorimotor processes, for example, in the discrimination of peripersonal space (i.e., portion of space within arm reach allowing manual interaction with objects) and extrapersonal space (i.e., space beyond reaching capacity; Rizzolatti et al., 1997, 2002; Gallese et al., 1999; Coello and Delevoye-Turrell, 2007; Gallese, 2007). These findings suggest that space may be represented by multiple sub-spatial maps partly delimited by body and action capabilities (Gross and Graziano, 1995; Rizzolatti et al., 1997). Therefore, it appears that the motor system not only plans and controls actions, but that the same neural processes appear to be involved in internal simulation of actions (Fadiga et al., 2000; Jeannerod, 2001), and that these simulations may be used to derive an embodied perception of space (Witt and Proffitt, 2008).

Space perception being delimited into different subspaces regarding action capacities has also been emphasized in neuropsychological (see Previc, 1998, for a review) and neuroimaging research (Weiss et al., 2000; Committeri et al., 2007; Quinlan and Culham, 2007; Brozzoli et al., 2012). Peripersonal space (for visuomotor interaction in reaching range) differs from three extrapersonal spaces (focal extrapersonal, action extrapersonal and ambient extrapersonal; for visual scanning and orientation in space); proposed to rely on different cortical networks (Previc, 1998). For example, bisecting lines in near space has been shown to activate dorsal visuomotor areas, whereas performing the same task on a more distant screen was shown to use ventral perceptual areas (Weiss et al., 2000). In addition to different subspaces, other neuroimaging studies have highlighted neural differences between the frames of reference taken by a participant (Committeri et al., 2004; Zaehle et al., 2007; Galati et al., 2010). The egocentric perspective (i.e., the location of an object from one’s own body; also termed body-referencing) involves a bilateral but mainly right-sided fronto-parietal network related to goal directed action planning (Vallar et al., 1999; Galati et al., 2000). The allocentric perspective (i.e., the location of an object relative to the location of another object or person) involves activation of similar areas of the dorsal stream to the egocentric perspective, though to a lesser extent, but also many additional areas in the ventral stream (for a review, see Galati et al., 2010). These observations suggest that egocentric perspective might recruit motor representations to a greater extent than the allocentric perspective. Consistent with these arguments, it has also been shown that the perception of reachable versus unreachable objects activates fronto-parietal networks (i.e., the precuneus and the parieto-occipital junction, the anterior parts of the cingulate gyrus and superior and medial frontal gyri, bilaterally) and the cerebellum, suggesting a contribution of dynamic motor representations facilitating the perceptive discrimination of peri- and extrapersonal spaces (Gallivan et al., 2009; Bartolo et al., 2014).

Visually determining whether an object is at a reachable distance is thought to rely on pre-reflective representations of body capacities for action (for a review, see Coello and Delevoye-Turrell, 2007). In reachability judgments tasks, it has been shown that perceived reaching limit can be influenced by the manipulation of action capability, with postural or environmental constrains (Carello et al., 1989; Rochat and Wraga, 1997; Fischer, 2000; Gabbard et al., 2007), height or position of the table where stimuli are presented (Carello et al., 1989), or participants wearing weights on the wrist (Rochat and Wraga, 1997). For example, hiding participants’ hands and providing them a biased visual feedback about the end-point location of their pointing movement has been used to modify a person’s perceived action capacity, and the manipulation has shown a moderation to perceived reachable space (Bourgeois and Coello, 2012). In a second example, a study used a motor constraint paradigm (i.e., by blocking the arms of participants) and showed response speed and accuracy interference for spatial localization decisions of stimuli within peripersonal space (Iachini et al., 2014). Further, physical (non-manipulated) differences in action capability such as handedness and visual laterality of target placement can also moderate reachability judgments (Fischer, 2005a; Gabbard et al., 2005a,b). Finally, motor disruption, for example through the use of transcranial magnetic stimulation (TMS) applied over the hand motor cortex of the left hemisphere, was shown to moderate response latencies in reachability judgments, particularly for stimuli positioned near the boundary of peripersonal space (Coello et al., 2008). Therefore, together, these effects demonstrate that moderations that normally influence action, also influence judgments of reachability, suggesting that reachability may be based on action representations that are constrained by the context in which the action could be performed (Fischer, 2000).

In parallel, studies focusing on the cognitive processes underlying distance perception have observed similar behavioral effects of action manipulation on distance estimation tasks (Proffitt, 2006; Witt, 2011). For instance, participants wearing a heavy backpack showed an increase in egocentric distance estimation compared to not wearing any backpack (Proffitt et al., 2003). Also, throwing a heavy compared to light ball to a target caused a subsequent greater estimation of the distance between the person and the same target (Witt et al., 2004). These two studies demonstrated that the manipulation of the effort associated with the action influenced space perception (Proffitt, 2006). In a further study, Witt and Proffitt (2008) added a concurrent ball squeezing task to the ball throwing task. It appeared that squeezing a rubber ball during distance estimation eliminated the influence of the heavy ball throwing, presumably through preventing ball throwing simulation (Witt and Proffitt, 2008). Distance estimation has also been investigated following the use of tools, understood to extend peripersonal space (Berti and Frassinetti, 2000; Farnè and Làdavas, 2000; Longo and Lourenco, 2006). For instance, after using a tool, participants perceived targets as closer than when no tool was used (Witt et al., 2005; Witt and Proffitt, 2008; Osiurak et al., 2012). Interestingly, when participants squeezed a rubber ball while making distance estimation judgments, the impact of tool use on distance estimation was reduced compared to making judgments without ball squeezing (Witt and Proffitt, 2008). As distance perception was moderated by tool use, and because the dual-task of ball squeezing reduced this moderation, it seems that motor simulation must provide a calibration metric for distance perception.

Altogether, these findings suggest that space perception benefits and is scaled to the representations of the body and its capacities. However, whether internal simulated actions do contribute to the perception of spatial distance is still an open question (Proffitt, 2013). The goal of the present study was to examine the contribution of action representations in both reachability and distance perception behaviors by investigating whether a concurrent motor task that may disrupt internal action representations would influence the perception of space. To assess this, participants completed three different spatial perceptual tasks (i.e., reachability, egocentric distance and allocentric length estimation) while performing concurrent hand (i.e., foam ball squeezing; Witt and Proffitt, 2008) or arm (i.e., weight lifting) actions in a within-participant design. With this manipulation, we tested whether similar interference of the concurrent actions would be observed in the reachability judgment task and the egocentric distance estimation task. We propose that these two tasks should show similar patterns of response time interference as, in previous studies, the manipulation of reach capacities influenced distance estimation (Witt and Proffitt, 2008; Osiurak et al., 2012; Morgado et al., 2013). In contrast, no dual-task moderation is expected in the allocentric length estimation task. Indeed, we argue that allocentric length estimation does not involve spatial localization relative to the body (or body referencing) and that action simulation processes are thus not recruited in this task. For the reachability judgment task, we predict a typical increase of response latency and error rate for targets placed near the boundary of peripersonal space (Gabbard et al., 2007; Bartolo et al., 2014). Moreover, an interaction between target location and dual-task for response latencies is expected in the reachability judgment task, with a stronger action dual-task effect for stimuli placed near the boundary of peripersonal space (Coello et al., 2008).

## Method

### Participants

There were 18 participants (aged between 18 and 25 years, *M* = 20.3, SD = 2.2, nine woman, three left-handed), all with normal or corrected-to-normal visual acuity and all naïve to the purpose of the experiment. The experiment was non-invasive and was approved by the ethics committee of the Institut de recherche en Sciences Psychologiques of the Université catholique de Louvain, in accordance with the ethical standards established by the Declaration of Helsinki.

### Apparatus, Stimuli, and Procedure

**FIGURE 1:**
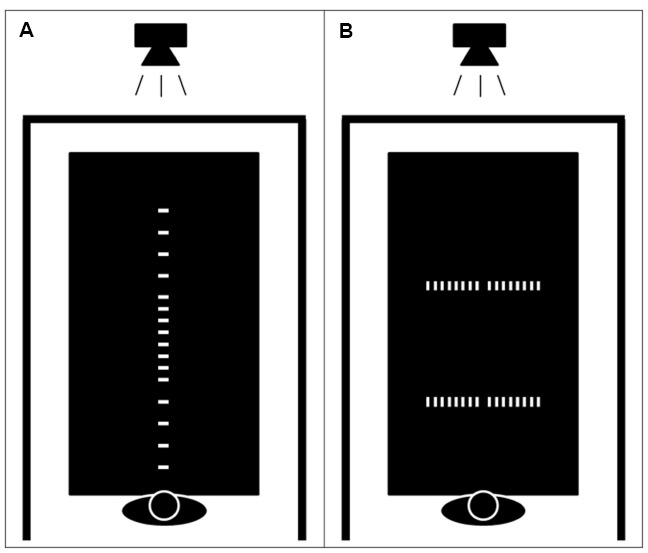
**Schematic representation of the experimental apparatus.** A projector placed above the table displayed the stimulus at various positions. Dark curtains surrounded the table in order to isolate the experimental environment from the rest of the room. Participants were seated on a fixed chair with their trunk touching the edge of the table. Possible locations of the stimuli in the reachability judgment and the egocentric distance estimation tasks **(A)**. Possible locations of the stimuli in the allocentric length estimation task **(B)**.

The apparatus consisted of a projector placed above a white table that was 215 cm long, 122 cm wide and 70 cm high. Black curtains surrounded the table in order to isolate the experimental environment from the rest of the room and reduce distractions. Participants sat on a chair situated in the middle of the small edge of the table and a microphone was placed above their head in order to record response latencies. The stimuli were composed of white rectangles (5 cm width and 2.5 cm length) displayed on a black background at various locations on the table (see Figure 1). A customized E-prime program (Schneider et al., 2002) was used to display the stimuli on the table and to control the experimental procedures.

All participants were required to perform three different tasks: reachability judgment, egocentric distance estimation, and allocentric length estimation. The reachability judgment and egocentric distance estimation tasks were made to the same stimuli. Rectangular shapes were projected on the tabletop at 16 different distances along the participant’s sagittal body-midline axis (35; 55; 65; 75; 80; 85; 87.5; 90; 92.5; 95; 100; 105; 115; 125; 145; and 165 cm), such that approximately half of the stimuli was placed within reach and the other half out of reach, with more closely spaced stimuli placed at the boundary of reach space. In the reachability judgment task, participants were asked to judge whether they could touch the stimuli displayed on the table without actually performing any reaching actions. They responded aloud “yes” if they thought that they could touch the rectangle, or “no” if they thought that the stimulus was out of reach. It was explicitly mentioned that they could imagine themselves leaning forward, but that their bottom could not leave the chair in their action simulation. Moreover, they were asked to keep their back against the chair backrest during the entire experiment. In the egocentric distance estimation task, participants were asked to estimate the distance in centimeters separating them from the rectangular stimulus. In the allocentric length estimation task, they estimated the distance separating two rectangles presented on the tabletop. The two rectangles were presented either at 70 or 130 cm from the participant (i.e., within and out of reach space), and there were eight possible lengths between the rectangles (8; 16; 24; 32; 40; 48; 56; and 64 cm). The two rectangles were always equidistant from the center of the table compared to the participant’s sagittal body mid-line. Within each task, the participants performed three different conditions of dual-task (run in separate trial blocks and counterbalanced within the tasks). Participants were either instructed to simply place their hands on the edge of the table (baseline condition), to perform arm actions (i.e., from fully laterally outstretch arm span to the flexion of elbows with the hands above the shoulders) with one-kilogram weights (arm action condition), or to perform foam ball squeezing hand actions placing their arms alongside their body (hand action condition). They were also trained with a metronome in order to perform the different actions at a specific rate (i.e., 40 per min).

Task order was counterbalanced across the participants. Each task consisted of three blocks of trials, each with a different dual-task condition (order of dual-tasks was also counterbalanced). Each block consisted of 16 different stimuli repeated four times, resulting in 64 randomized trials per block. Each trial started with a beep sound lasting 700 ms, then a stimulus displayed until participants responded, and finally, a blank screen for 1000 ms. In all tasks, the participant had to respond as fast as possible while keeping errors to a minimum. Each task started with a small practice session to make sure that participants fully understood the instructions and experimental set up, and that they performed the different dual-task actions at a specific frequency paced by a metronome. At the end of the experiment, the experimenter measured the height of participant’s eyes, the length of their arms (from neck base to the edge of the middle finger) and their actual reaching limit (the participants furthest reach while being seated on the chair).

### Data Analyses

For the reachability judgment and egocentric distance estimation tasks, the 16 distances were averaged into four different distance categories (i.e., very close with distances 35; 55; 65; 75; close with distances 80; 85; 87.5; 90; far with distances 92.5; 95; 100; 105; and very far with distances 115; 125; 145; 165). Repeated measures analyses of variance (ANOVAs) were conducted with distance categories and dual-task conditions (i.e., no actions; arm actions or hand actions) as within-subject factors. For the allocentric length estimation task, two length categories were formed from the eight different stimuli (short: 8; 16; 24; 32; long: 40; 48; 56; 64). Repeated measures ANOVAs were conducted with length categories, the position from the participant (70 cm vs. 130 cm) and the dual-task conditions as within subject factors. The dependent variables were response latency and accuracy. For the reachability judgment, accuracy was computed on the basis of the participant’s real reach capability measured at the end of experiment. For each stimuli distance, a trial was considered as an error when participants under-estimated (i.e., responded that they could not reach a target when they could) or over-estimated (i.e., responded that they could reach a target when they could not) their reachability. To assess accuracy for egocentric distance and allocentric length estimations independently of the magnitude of the target to be estimated, an error rate was computed by subtracting the actual distance from the participant’s response and dividing this difference by the actual distance (i.e., [(participant’s response – actual distance)/actual distance]; where a positive value indicates overestimation, a negative value indicates underestimation, and a value of 0 means perfect accuracy for a similar index, see Crollen et al., 2013).

Bonferroni correction (*_BC_*) was applied where multiple *post hoc* comparisons were used. Data from unreliable trials (no response or microphone failures), and outlier responses (on which response latency was above or below 2.5 standard deviations from the overall mean) were excluded from the analyses. This led to the removal of 2.6, 1.0, and 4.2% of unreliable trials, and 2.8, 2.3, and 1.3% of outlier response latencies from the total trials of the reachability judgment, egocentric distance estimation and allocentric length estimation tasks respectively.

## Results

### Reachability Judgment Task

**FIGURE 2:**
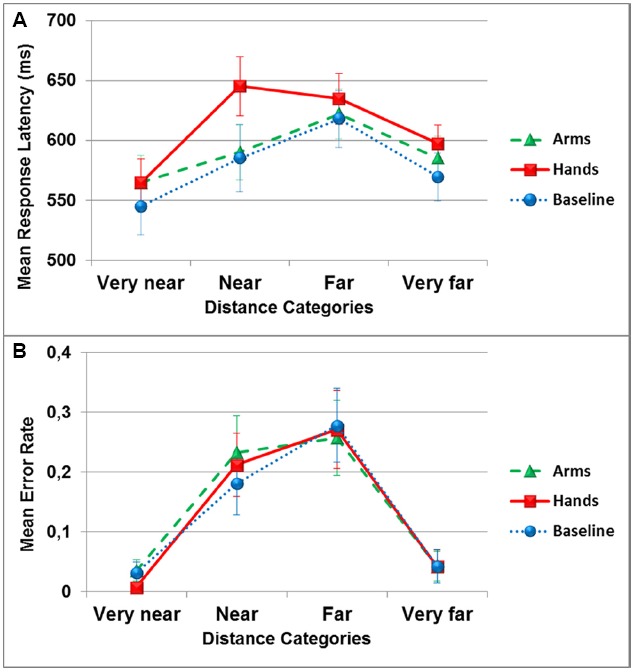
**Mean response latency in milliseconds (A) and mean error rate (B) in the reachability judgment task as a function of distance categories and dual-task conditions (circles: no action, triangles: arm actions, squares: hand actions).** Error bars represent one Standard Error of the Mean (SEM).

The analysis of response latency revealed a significant main effect of the dual-task conditions [*F*(2,34) = 3.5, *p* < 0.05], with a significant difference between the hand action condition (*M* = 610 ± 76) and the no action condition (*M* = 579 ± 93; *p_BC_* < 0.05, ${\text{η}}_{p}^{2}$ = 0.17). A significant main effect of the distance categories was observed [*F*(3,51) = 10.1, *p* < 0.001, ${\text{η}}_{p}^{2}$ = 0.37], with the very near (*M* = 558 ± 90) and very far (*M* = 583 ± 71) categories not being significantly different from each other (*p_BC_* > 0.05), but significantly different from the near (*M* = 606 ± 98) and far (*M* = 624 ± 82) categories (all *p_BC_* < 0.05). The difference between the near and far categories wasn’t significant (*p_BC_* > 0.05). The interaction between the two variables was significant [*F*(6,102) = 3.6, *p* > 0.01, ${\text{η}}_{p}^{2}$ = 0.17]. Separate ANOVAs were run for each distance category with the dual-task condition as factor. A significant effect of the dual-task was only observed in the near distance category [*F*(2,34) = 7.3, *p* < 0.01, ${\text{η}}_{p}^{2}$ = 0.30], and pairwise comparisons showed that the hand action condition was significantly different from the arm action and the no action conditions (*p_BC_* < 0.05; see Figure 2A).

The analysis of accuracy (i.e., absolute error, irrespective of over or underestimation of reach) showed a significant main effect of distance categories [*F*(3,51) = 8.5, *p* < 0.001, ${\text{η}}_{p}^{2}$ = 0.33], with the very close (*M* = 0.024 ± 0.042) and the very far (*M* = 0.042 ± 0.11) distances significantly different from the close (*M* = 0.21 ± 0.19) and far (*M* = 0.26 ± 0.24) distances (all *p_BC_* < 0.05). Participants made more errors for distances situated on the boundary between reachable and unreachable space than for the very close or very far distances. There was no main effect of the dual-task conditions [*F*(2,34) = 0.12, *p* > 0.05, ${\text{η}}_{p}^{2}$ = 0.007], and no interaction between distance categories and dual-task conditions [*F*(6,102) = 1, *p* > 0.05, ${\text{η}}_{p}^{2}$ = 0.03; see Figure 2B].

### Egocentric Distance Estimation Task

**FIGURE 3:**
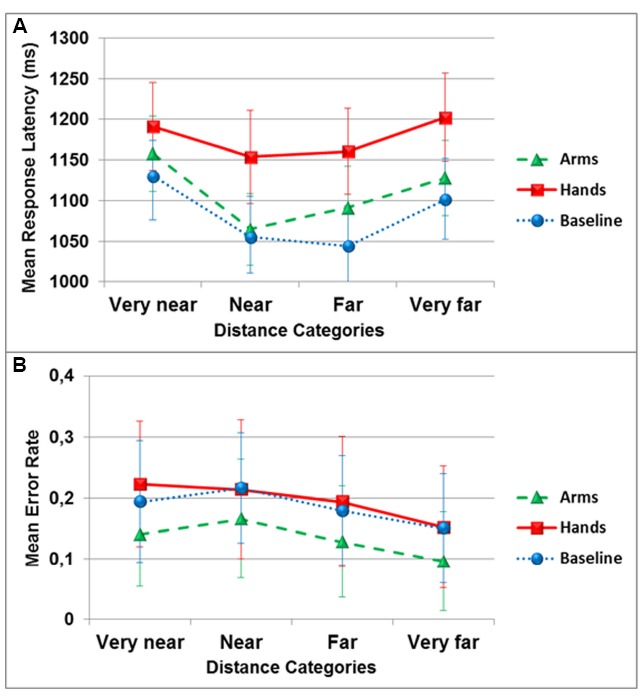
**Mean response latency in milliseconds (A) and mean error rate (B) in the egocentric distance estimation task as a function of distance categories and dual-task conditions (circles: no action, triangles: arm actions, squares: hand actions).** Error bars represent 1 Standard Error of the Mean (SEM).

The ANOVA on response latencies revealed a significant main effect of the dual-task condition [*F*(2,34) = 9.5, *p* < 0.01, ${\text{η}}_{p}^{2}$ = 0.36], with the hand action condition (*M* = 1176 ± 221) significantly different from the no action condition (*M* = 1082 ± 195; *p_BC_* < 0.01). A significant main effect of the distances was also observed [*F*(3,51) = 6.3, *p* < 0.01, ${\text{η}}_{p}^{2}$ = 0.27], with the very near category (*M* = 1159 ± 196) significantly different from the near category (*M* = 1091 ± 197; *p_BC_* < 0.05). The two variables did not interact [*F*(6,102) = 0.87, *p* > 0.05, ${\text{η}}_{p}^{2}$ = 0.05; see Figure 3A].

The analysis of accuracy revealed no effect of the dual-task conditions [*F*(2,34) = 2.12, *p* = 0.15, ${\text{η}}_{p}^{2}$ = 0.11], of the distance category [*F*(3,51) = 2.9, *p* = 0.069, ${\text{η}}_{p}^{2}$ = 0.15], and no interaction [*F*(6,102) = 0.36, *p* > 0.05, ${\text{η}}_{p}^{2}$ = 0.02; see Figure 3B].

### Allocentric Length Estimation Task

**FIGURE 4:**
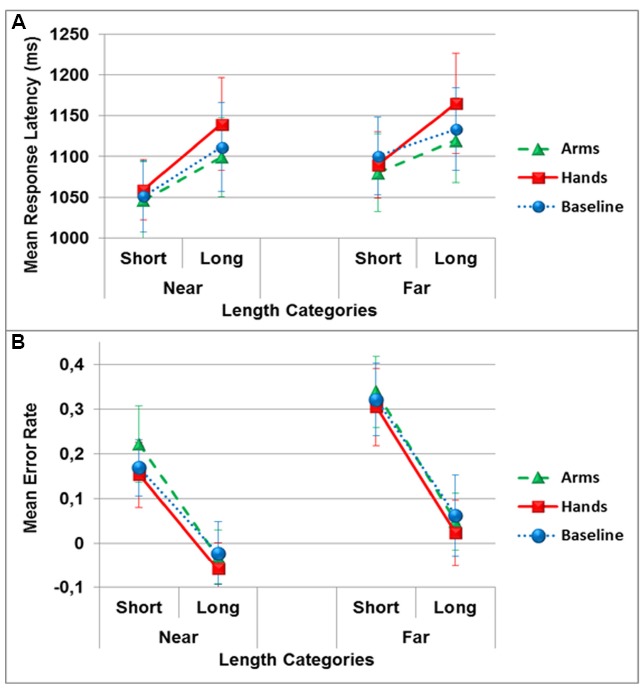
**Mean response latency in milliseconds (A) and mean error rate (B) in the allocentric length estimation task as a function of distance categories and dual-task conditions (circles: no action, triangles: arm actions, squares: hand actions).** Error bars represent one Standard Error of the Mean (SEM).

The ANOVA of the response latencies did not reveal a significant main effect of the dual-task conditions [*F*(2,34) = 0.8, *p* > 0.05, ${\text{η}}_{p}^{2}$ = 0.04]. There was however, a significant main effect of the position [*F*(1,17) = 10.8, *p* < 0.01, ${\text{η}}_{p}^{2}$ = 0.39] indicating that lengths positioned near (*M* = 1084 ± 186) the participants were responded to faster than lengths presented further away (*M* = 1114 ± 195). There was also a significant main effect of length category [*F*(1,17) = 9.9, *p* < 0.01, ${\text{η}}_{p}^{2}$ = 0.37], with participants responding faster to shorter (*M* = 1071 ± 172) than longer (*M* = 1127 ± 216) lengths. There were no significant interactions (see Figure 4A).

The analysis of accuracy showed no significant main effect of dual-task conditions [*F*(2,34) = 0.76, *p* > 0.05, ${\text{η}}_{p}^{2}$ = 0.04], but a significant main effect of the position [*F*(1,17) = 38,9, *p* < 0.001, ${\text{η}}_{p}^{2}$ = 0.69], with the lengths appearing near (*M* = 0.072 ± 0.25) showing a smaller overestimation bias than lengths appearing far (*M* = 0.18 ± 0.30) from participants. There was also an effect of length categories [*F*(1,17) = 30.3, *p* < 0.001, ${\text{η}}_{p}^{2}$ = 0.64], with short lengths (i.e., 8; 16; 24; 32 cm; *M* = 0.252 ± 0.31) showing a larger overestimation bias than long lengths (i.e., 40; 48; 56; 64 cm; *M* = 0.004 ± 0.28). The only significant interaction observed was the one between positions and length categories [*F*(1,17) = 4.9, *p* < 0.05, ${\text{η}}_{p}^{2}$ = 0.22]. Accuracy differences between short and long lengths was significantly smaller in the near compared to far position [*t*(17) = 2.2, *p* < 0.05; see Figure 4B].

## Discussion

The perception of space is thought to benefit from the ability to mentally represent action (Rizzolatti et al., 1997; Gallese, 2007; Witt and Proffitt, 2008), and to use action capability as an index for understanding how objects are positioned relative to ourselves (Coello and Delevoye-Turrell, 2007; Witt and Proffitt, 2008; Proffitt, 2013). As many findings show similarities between action execution and action simulation (Decety et al., 1989; Parsons, 1994; Hanakawa et al., 2008), an interference of action execution on action simulation and beyond it, on space processing, is expected. However, it is currently unclear if reachability judgment (whether an object is in reach) and egocentric distance estimation (the distance between the viewer and the object) rely on the same body capability representations and action simulation processes. The aim of the present study was to evaluate whether performing a motor dual-task interfering with motor simulation processes would moderate space perception (extending dual-task effects to distance estimation; Witt and Proffitt, 2008). Furthermore, within the same participants, we wanted to determine for the first time whether two commonly used measures of space perception (reachability judgment and egocentric distance estimation) were based on a common action simulation mechanism.

For the reachability judgment task, significantly slower and less accurate responses were observed for targets presented close to the boundary between reachable and unreachable spaces, in comparison to the distances further from the boundary (very near and very far). This finding is in accordance with other studies investigating reachability judgment (e.g., Fischer, 2005b; Gabbard et al., 2005a, 2007; Bartolo et al., 2014). Furthermore, the dual-task conditions only affected response latencies, where performing foam ball squeezing actions significantly increased response latencies compared to the arm action and the control condition. This was particularly the case for judgments to targets placed in peripersonal space and near the boundary between peri- and extrapersonal spaces. This observation extends the finding that TMS application to the hand associated motor cortex slowed down participants perceptual judgments of whether a target was reachable or not, with a greater disruption when the targets appeared at the boundary of peripersonal space (Coello et al., 2008). Therefore, using the current method, we support the argument that when performing visually based judgments of whether a target is reachable, internal motor representations appeared to be recruited rather than being an epiphenomenal consequence of the reachability judgment (Coello and Delevoye-Turrell, 2007; Bartolo et al., 2014).

For egocentric distance estimation, participants tended to respond faster to targets positioned near compared to very near, but there was no influence of target position on accuracy. Although the influence of distance on the speed of response had a different profile than the one observed in the reachability judgment task, the ball-squeezing dual-task again slowed participant’s responses relative to the no-action condition, but showed no interaction between target distance and the dual-task condition. This result supports the idea that motor simulation processes are involved in perceiving egocentric distances of objects (Witt et al., 2004; Proffitt, 2006, 2013; Witt and Proffitt, 2008), and that the simulation of reach capability may serve as a calibration metric for distance perception scaling spatial locations in the environment relative to body and its capacities (Witt et al., 2005; Linkenauger et al., 2009; Osiurak et al., 2012; Morgado et al., 2013).

In opposition to reachability judgment and egocentric distance estimation, allocentric length estimation was not influenced by the concurrent actions. This suggests that allocentric length estimation does not rely on motor simulation processes or body representation. An effect of stimulus magnitude was observed with participant’s overestimating short compared to long lengths. This effect can be attributed to a contraction bias described in the literature, where participants have their magnitude estimations pulled toward the stimuli range center, leading to the underestimation of large and the overestimation of small stimuli (Poulton, 1979). Additionally, an effect of position was observed, where participants made faster and more accurate responses to targets positioned near compared to far from them. More specifically, participants reported far positioned lengths as being longer than near positioned lengths despite the fact that the stimuli were identical in length. This effect may be due to a compensation of size constancy in depth where objects presented further from participants appear smaller (Gregory, 1963). This effect could also be due to a Ponzo illusion (Ponzo, 1912), where identical lines are perceived as different when placed within a triangle or a trapezoid shape (Fisher, 1968). In our experimental set up, even if the table was rectangular, it appeared as a trapezium for participants (i.e., near edge is visually larger than the far edge of the table) perhaps causing Ponzo illusion-like effect on the stimuli in this task.

Interestingly, only the ball squeezing dual-task interfered with reachability judgment and egocentric distance estimation. Although one might have expected that the concurrent arm movement would have had a similar disruptive effect, this was not the case. An interpretation of this interaction effect could be the manner that reaching actions might be internally simulated or represented. The ball squeezing actions specifically required acting on an object rather than just moving the object continuously as in the arm action dual-task. We propose that the object or goal directed actions may be more interfering, involving both reach and grasp integrated representations (Jeannerod, 1997; Paulignan et al., 1997). Perhaps similarly, the effect could be explained by the theory of event coding (TEC) framework for interactions between perception and action (Hommel et al., 2001; Hommel, 2009). According to TEC, perceived events and action intentions/goals (or to be produced events) are coded within a common representational medium of distal events. Perception thus includes action planning or simulation that takes into account the goals an individual has regarding a distal event (i.e., the intended change to be performed). In the present study, the ball-squeezing dual-task required participants to act upon an object with the intention of modifying the object structure, generating an object-directed distal event. As the target stimuli in the reachability and distance estimation tasks were also processed as distal events, the two different events would have had to be represented simultaneously, and this competition in representation might have caused interference in event coding processes leading to longer response latencies. Moreover, for goal directed grasping in peripersonal space, the representation of the object position had to be coded in hand-centered coordinates rather than regarding arm position (Brozzoli et al., 2012). Experiments have shown that multisensory coding of peripersonal space (measured with cross-modal visual-tactile interactions) was particularly influenced by object oriented grasping actions (Brozzoli et al., 2009, 2010). Their results showed that cross-modal congruency effects were stronger during action execution compared to a static condition (Brozzoli et al., 2009) and that this congruency effect was stronger during the execution of grasping action compared to pointing (reaching) action (Brozzoli et al., 2010). If the perception of peripersonal space was equally considered to be based on hand-centered simulation processes, then only the squeezing dual-task would influence space perception, and not the arm action dual-task condition.

From the common finding of the dual-task effects on both reachability judgment and egocentric distance estimation, we argue that egocentric space processing requires motor simulation processes where the viewer evaluates the position of a target in relation to their body and simulate an action within their capacity. This finding is consistent with several fMRI studies showing that, in egocentric perceptual tasks (e.g., judging which of two objects are closest to the viewer), there was a greater activation of a fronto-parietal network including the posterior parietal cortex and premotor areas (Committeri et al., 2004; Galati et al., 2010). These brain areas are used for action planning processes and, furthermore, are shown to be active when participants imagine executing actions (Hanakawa et al., 2008; Macuga and Frey, 2012). This is in contrast to allocentric perceptual tasks (e.g., judging which of two objects are closest to a third one) that show activations scattered across both the ventral and dorsal areas (Committeri et al., 2004; Galati et al., 2010), suggesting that allocentric length estimation may require different cognitive processes not related to action simulation.

In the present study, the effect of the dual-task was limited to response latency effects. Our analyses showed that the dual-task conditions had no influence on actual reachability judgments or distance estimates. This finding is consistent with previous dual-task experiments (Witt and Proffitt, 2008), where it was reported that squeezing soft balls at the same time as estimating the distance of targets showed no moderation of the reported distances when no tool was present. This suggests that represented body metrics are not modified by the concomitant execution of actions, as no extension or reduction of perceived peripersonal space was observed. Another interesting finding was that the response latency of the ball squeezing dual-task differed for reachability judgment and egocentric distance estimation. For reachability judgment, responses were slowed specifically for targets in the peripersonal space whereas the dual-task slowed responses to all target positions for egocentric distance estimation. These two findings suggest that while common motor resources are recruited for the two tasks, the manner in which those motor resources are used might be different. For reachability judgments, responses are particularly slowed for peripersonal targets close to the boundary between peripersonal and extrapersonal space. This could be explained through inefficiency or difficulty in the use of action simulation when at the limit of the participant’s capability. For egocentric distance estimation however, the perceptual response latencies were similar for all targets, irrespective of whether they were within or outside of reach.

In conclusion, our study supports the idea that internal representations of action contribute to the perception of external space (Coello and Delevoye-Turrell, 2007; Witt and Proffitt, 2008; Bartolo et al., 2014). Visually determining what is reachable engages the simulation of a motor act that can be interfered with a hand motor dual-task. Moreover, we find that similar internal simulations of reach might also serve as a metric for egocentric distance perception. Therefore, reachability and egocentric distance perception appear to be linked (Osiurak et al., 2012; Morgado et al., 2013), requiring overlapping reaching simulation cognitive processes. However, despite the finding that similar motor resources appeared to be recruited for both behaviors, it could be that these resources are used differently for each behavior. This motor contribution to egocentric space perception may require body referencing, and we argue that in allocentric space, no such motor contribution is recruited. These findings suggest that in order to perceive the environmental layout surrounding a person, the viewer not only represents perceived space from sensorial inputs, but they also simulate the potential body and action interactions within space, supporting an embodied view of space perception (Coello and Delevoye-Turrell, 2007; Proffitt, 2013).

### Conflict of Interest Statement

The authors declare that the research was conducted in the absence of any commercial or financial relationships that could be construed as a potential conflict of interest.
